# A Novel Method of Newborn Chest Compression: A Randomized Crossover Simulation Study

**DOI:** 10.3389/fped.2018.00159

**Published:** 2018-05-29

**Authors:** Jacek Smereka, Lukasz Szarpak, Jerzy R. Ladny, Antonio Rodriguez-Nunez, Kurt Ruetzler

**Affiliations:** ^1^Department of Emergency Medical Service, Wrocław Medical University, >Wrocław, Poland; ^2^Department of Emergency Medicine, Medical University of Warsaw , Warsaw, Poland; ^3^Department of Emergency Medicine and Disaster, Medical University of Białystok, Białystok, Poland; ^4^Clinursid Research Group, School of Nursing, University of Santiago de Compostela, Santiago de Compostela, Spain; ^5^Department of General Anesthesiology, Anesthesiology Institute, Cleveland Clinic, Cleveland, OH, United States

**Keywords:** chest compression, technique, cardiopulmonary resuscitation, newborn, physicians

## Abstract

**Objective:** To compare a novel two-thumb chest compression technique with standard techniques during newborn resuscitation performed by novice physicians in terms of median depth of chest compressions, degree of full chest recoil, and effective compression efficacy.

**Patients and Methods:** The total of 74 novice physicians with less than 1-year work experience participated in the study. They performed chest compressions using three techniques: (A) The new two-thumb technique (nTTT). The novel method of chest compressions in an infant consists in using two thumbs directed at the angle of 90° to the chest while closing the fingers of both hands in a fist. (B) TFT. With this method, the rescuer compresses the sternum with the tips of two fingers. (C) TTHT. Two thumbs are placed over the lower third of the sternum, with the fingers encircling the torso and supporting the back.

**Results:** The median depth of chest compressions for nTTT was 3.8 (IQR, 3.7–3.9) cm, for TFT−2.1 (IQR, 1.7–2.5) cm, while for TTHT−3.6 (IQR, 3.5–3.8) cm. There was a significant difference between nTTT and TFT, and TTHT and TFT (*p* < 0.001) for each time interval during resuscitation. The degree of full chest recoil was 93% (IQR, 91–97) for nTTT, 99% (IQR, 96–100) for TFT, and 90% (IQR, 74–91) for TTHT. There was a statistically significant difference in the degree of complete chest relaxation between nTTT and TFT (*p* < 0.001), between nTTT and TTHT (*p* = 0.016), and between TFT and TTHT (*p* < 0.001).

**Conclusion:** The median chest compression depth for nTTT and TTHT is significantly higher than that for TFT. The degree of full chest recoil was highest for TFT, then for nTTT and TTHT. The effective compression efficiency with nTTT was higher than for TTHT and TFT. Our novel newborn chest compression method in this manikin study provided adequate chest compression depth and degree of full chest recoil, as well as very good effective compression efficiency. Further clinical studies are necessary to confirm these initial results.

## Introduction

Cardiac arrest in newborns and infants is a rare emergency situation, but requires highly skilled healthcare providers to perform high quality chest compressions (1). Some newborns may need resuscitation at birth and only a small part require chest compression after proper airway management and ventilation. Chest compression quality plays a crucial role in generating perfusion to vital organs in newborns and infants, affecting survival and neurological outcome after cardiac arrest (2). European Resuscitation Council (ERC) and American Heart Association (AHA) recommend high chest compression quality in all age groups as the key element in cardiopulmonary resuscitation (CPR) (3, 4).

There are two main chest compression techniques in newborns—two thumbs encircling the torso and supporting the back of the newborn (TTHT) and the two-finger technique (TFT). Several studies indicate that TTHT generates better chest compression rate and depth, blood pressure, and coronary perfusion pressure than TFT (5–7). The current guidelines for infant CPR suggest TFT for one-rescuer settings and TTHT for two rescuers for infant chest compressions (6, 8). It should be taken into account that TTHT can pose difficulties in ventilation-compression coordination in one-rescuer settings. In 2015, CoSTR guidelines stated that TTHT was the preferred infant chest compression method compared with TFT (9, 10).

Although TTHT is regarded as a standard chest compression technique in newborns and infants, the main disadvantage of this method is the issue of inadequate size of the rescuer hand and muscle strength between the thumb and 4 fingers, which limits the power necessary to generate enough force.

In addition to the above described standard techniques, there are also some modifications, including the two-thumb technique developed by Smereka et al. (11–14) and some techniques developed by other authors (1, 15).

**Figure 1:**
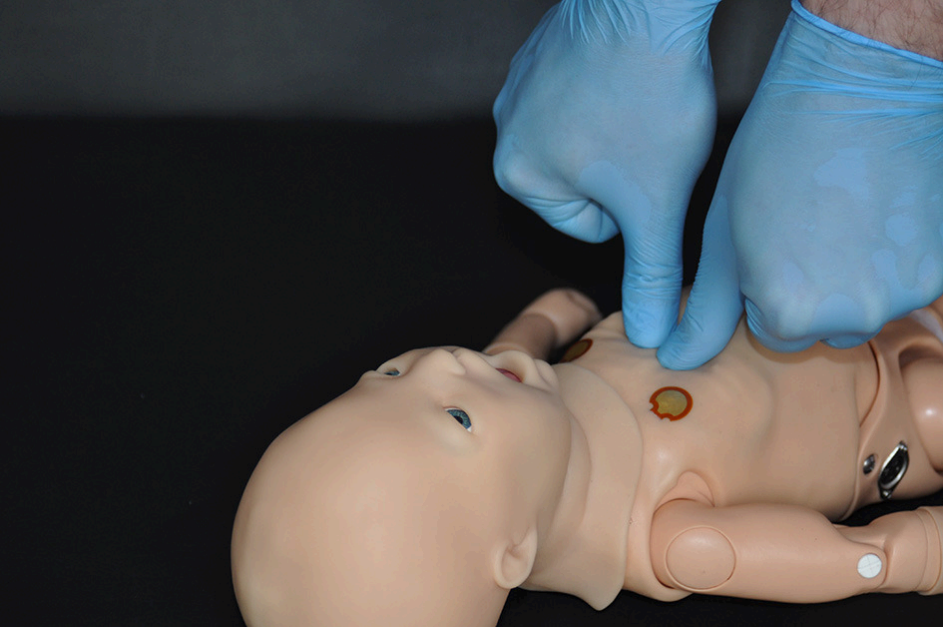
The new newborn chest compression technique.

The method developed by Smereka et al. (nTTT, new two-thumb technique; SupplementaryVideo 1) consists in directing two thumbs at the angle of 90° to the chest while closing the fingers of both hands in a fist (Figure 1). The thumbs remaining in the same line with arms can enable better chest compression with less fatigue (well-known phenomenon for both TTHT and TFT) for the rescuer and generate higher force compared with TFT. In the TFT technique, the chest compression quality depends on finger and hand strength of the rescuer, and this phenomenon is significantly limited in the nTTT method.

The initial results, described in previous studies by Smereka and Szarpak, suggest that nTTT can facilitate obtaining adequate chest compression depth and rate, as well as a full chest recoil and other hemodynamic parameters and can be superior compared with the standard TFT and TTHT techniques. This study is a continuation of research described earlier (11–14).

The aim of the current study was to compare the novel two-thumb chest compression technique with the standard techniques during newborn CPR performed by novice physicians in terms of median depth of chest compressions, degree of full chest recoil, and effective compression efficacy.

## Methods

### Study design

This was a randomized, crossover, observational study. It was approved by the institutional review board of the Polish Society of Disaster Medicine (Approval No. 251.11.2017.IRB), and informed consent was provided by each participant. The study is a continuation of a series of studies on the evaluation of a new technique of chest compressions in infants and newborns (11, 12, 14, 16).

### Chest compression techniques

The participants performed chest compressions with the use of 3 techniques:

1. The nTTT technique. The novel method of chest compressions in an infant consists in using two thumbs directed at the angle of 90° to the chest while closing the fingers of both hands in a fist.
2. TFT, previously a standard method for infant chest compression. With this method, the rescuer compresses the sternum with the tips of two fingers.
3. TTHT. In this technique, two thumbs are placed over the lower third of the sternum, with the fingers encircling the torso and supporting the back. The method was associated with better coronary artery perfusion and less rescuer fatigue than TFT (11).

### Training

Prior to the training, independent instructors demonstrated the correct performance of the 3 chest compression techniques investigated in the study. After the demonstration, the participants took part in a practical training which included performing a 2-min cycle of CPR. During the training, a SimBaby® (Laerdal, Stavanger, Norway) training manikin was used to represent an infant. The participants did not receive any vocal or written feedback regarding their performance during the study period.

### Evaluation

**Figure 2:**
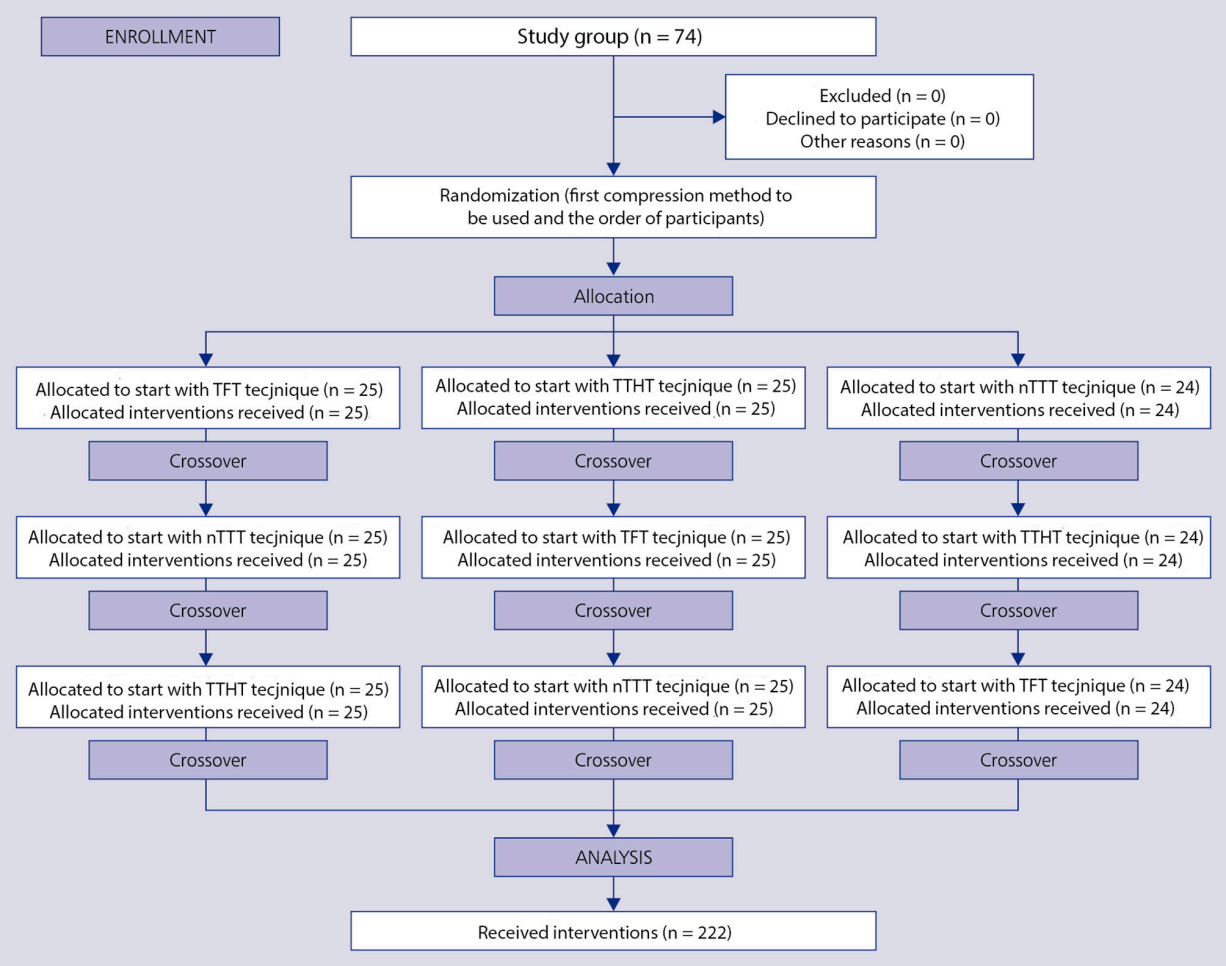
Consolidated Standards of Reporting Trials (CONSORT) flow diagram.

A week after the theoretical and practical training, the study participants proceeded to the testing part of the study. The order of both the participants and the applied chest compression techniques was randomized. For this purpose, the Research Randomizer software was used (Figure 2). During the testing part, chest compressions were performed on a Tory® S2210 Tetherless and Wireless Full-term Neonatal Simulator (Gaumard Scientific, Miami, FL, USA) that continuously records compression depth, compression rates, chest full release, and the rate of effective compressions.

The manikin used in the testing part simulates an approximately 2.7-kg, 40-week term newborn and is designed to serve as a realistic aid for learning newborn CPR. In order to rule out the impact of the rescuer's growth on the newborn chest compression quality, the simulator was placed on a gel mattress at a height adjusted to the iliac crest of each rescuer for standardization. Data were continually recorded electronically for the subsequent analysis. The monitoring screen was not visible for the participants, and no feedback was given. The participants used headsets, in which they heard a metronome set at the frequency of 100/min. In this way, it was possible to standardize the parameters of chest compressions and focus on the quality of compressions measured on the basis of their depth and the degree of chest relaxation (14). Full chest recoil was measured by the manikin sensors and software, and defined as the optimal situation when no force is applied to the chest at the end of chest decompression, so the next chest compression cycle begins with no force applied to the chest. Each CPR session lasted for 2 min and the participants were able to rest between the application of the 3 different chest compression techniques for at least 5 min.

Subjective self-assessment of the performance was recorded at the end of each participant's study period. Moreover, the study participants also evaluated the chest compression methods for fatigue, using a 10-point scale (1–minimum fatigue, 10–a very tiring technique).

### Statistical analysis

Data were analyzed with the statistical package Statistica 13.3 (TIBCO Software Inc., Tulsa, OK, USA). Continuous and original data are presented as median and interquartile range (IQR), and the categorical data are presented as raw numbers and frequencies. Non-parametric tests were used because the data distribution was not normal, as implied by the Shapiro-Wilk and Kolmogorov-Smirnov tests. Values of *p* < 0.05 were considered significant.

## Results

The total of 74 participants were enrolled in the trial. All of them were physicians (residents) with less than 1-year clinical experience and took part in the study on a voluntary basis. The demographic data for the study participants are shown in Table 1.

**Table 1:** Demographic characteristics of the participants.

**Parameter**	**Value**	**Median (IQR)**
Age, years	74	25 (24.5–26)
Sex, n (%)		
Male	42 (57%)	
Female	32 (43%)	
Body mass index, kg m^−2^	74	22.5 (20–24.5)

The depth of chest compressions with the 3 techniques applied is shown in Figure 2. The median depth of chest compressions for nTTT was 3.4 cm (IQR, 3.4–3.6) cm, for TFT−2.5 (IQR, 2.2–2.6) cm, and for TTHT−3.2 (IQR, 3.2–3.5) cm.

**Figure 3:**
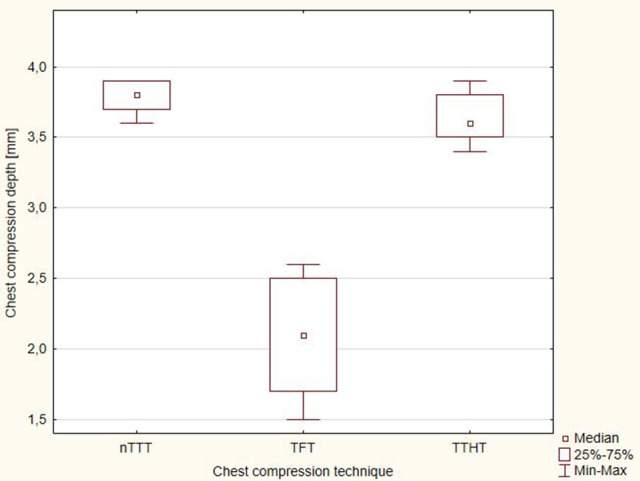
The distribution of chest compression depth with time.

The distribution of chest compression depth with time is presented in Figure 3. There was a statistically significant difference between nTTT and TFT, and TTHT and TFT (*p* < 0.001) for each time interval during CPR (Supplementary Table).

**Figure 4:**
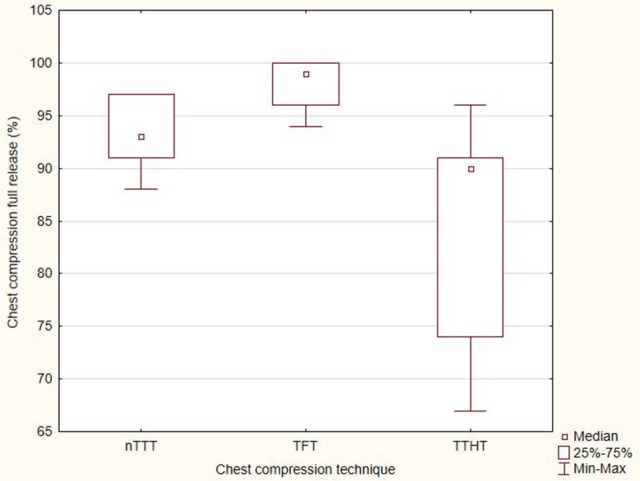
The percentage of chest compression full release.

The degree of full chest recoil in the tested techniques was varied and amounted to 93% (IQR, 91–97) for nTTT, 99% (IQR, 96–100) for TFT, and 90% (IQR, 74–91) for TTHT. There was a statistically significant difference in the degree of complete chest relaxation between nTTT and TFT (*p* < 0.001), nTTT and TTHT (*p* = 0.016), and TFT and TTHT (*p* < 0.001). A graphical summary of the percentage of chest compression full release is presented in Figure 4.

**Figure 5:**
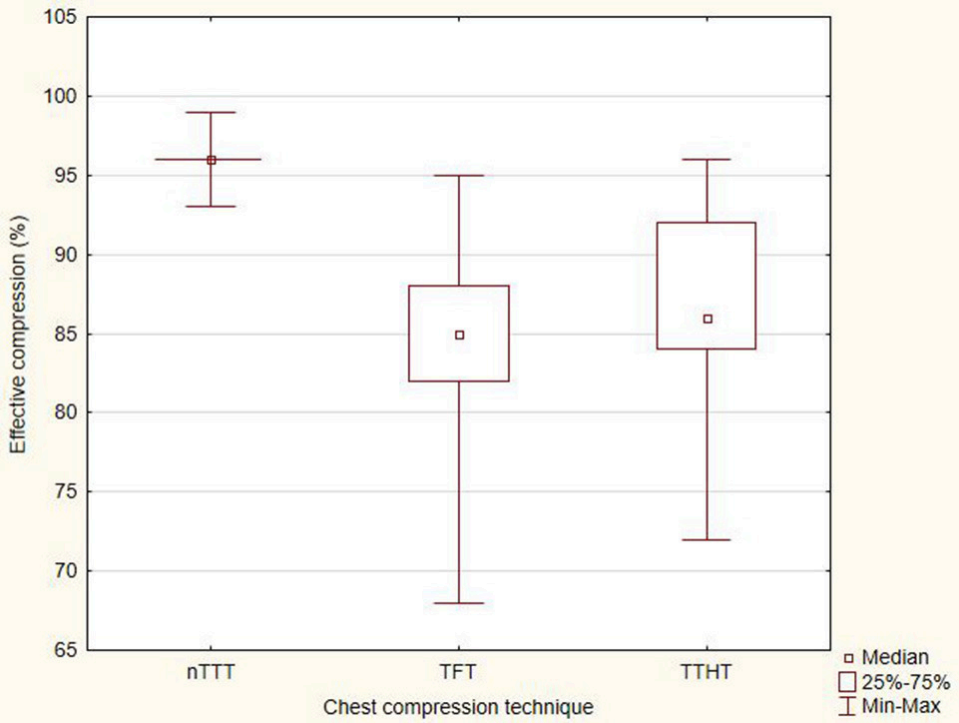
The percentage of effective compression.

The compression efficiency with the use of nTTT equaled 96% (IQR, 96–96). When the TFT method was used, the compression efficiency achieved 85% (IQR, 82–88), while in the case of TTHT−86% (IQR, 84–92). The above results showed statistically significant differences between nTTT and TFT (*p* < 0.001) and between nTTT and TTHT (*p* < 0.001) (Figure 5).

The study participants indicated nTTT as the most preferred method of chest compressions (58.1%), followed by TFT (36.5%), while TTHT was pointed at by 5.4%. Rescuer fatigue in the case of the tested methods was differentiated and amounted to 2.5 (IQR, 1.5–2.5) points for nTTT, 5.5 (IQR, 4–6) points for TFT, and 5.5 (IQR, 4.5–7) points for TTHT.

## Discussion

This was the first study evaluating the new method of chest compression in newborns with the use of a metronome as a tool to determine the frequency of chest compressions. This form of study allowed to standardize the frequency of chest compressions; therefore, it was possible to minimize errors in chest compression depth and relaxation resulting from different chest compression rates (12, 17, 18). Our results suggest that the nTTT technique could generate a better compression depth and better chest recoil during newborn CPR performed by novice physicians.

In accordance with the current guidelines for CPR published by the AHA (19), as well as by ERC (8), the elements affecting the quality of chest compressions and thus the quality of CPR are: the frequency of chest compressions, depth of compression, complete chest recoil, and correct positioning of the hands on the chest.

The optimal method of chest compressions recommended by AHA in the case of CPR conducted by one rescuer is TFT, and in the case of two rescuers—TTHT. These have been compared in animal and simulation models of cardiac arrest and experimental designs for systolic and diastolic blood pressures, coronary perfusion pressures (6, 7, 20). The study published by Christman et al. (21), who conducted research involving physicians and neonatal nurses, demonstrated that the TTHT technique was superior to TFT, allowing to achieve greater depth and less variability which each compression. Also, Houri et al. (5), in their study on an swine weighing 10 kg, proved that TTHT produced significantly higher systolic blood pressure. Jiang et al. (10) revealed that TTHT resulted in chest compressions more than 0.5 cm deeper than in the case of TFT. Our study showed that the depth of chest compressions with the nTTT technique equaled 3.8 cm and was definitely bigger when compared with the standard TFT technique (2.5 cm), which confirms that the TFT method is less effective than TTHT and nTTT in the context of obtaining adequate chest compression depth.

The quality of chest compression, beyond the depth and frequency, is also affected by the degree of complete chest recoil after each compression (22–24). The chest compression at the appropriate depth and then allowing the chest to fully recoil is the key element influencing the pressure difference in the chest and thereby enabling organ perfusion. In the current study, the degree of complete chest recoil varied among nTTT, TFT, and TTHT and amounted to 93 vs. 99 vs. 90%, respectively. These results are also confirmed by other studies (11–14, 25), in which chest compressions in a newborn or infant performed with the TFT technique were associated with a higher degree of chest relaxation compared with the standard two-thumbs encircling hands technique. The standard chest compression rate and ventilation rate for newborns at birth is 3:1 (8) and allows to achieve approximately 90 chest compressions per minute. On the other hand, the standard chest compression rate in pediatric life support is 100–120 per minute. Our study concentrated on chest compression quality only to compare different techniques of chest compression. The 100 chest compression per minute rate was chosen deliberately to check the chest compression quality among newborns.

## Study limitations

The presented study had several limitations. The first is the fact that the results were obtained on the basis of simulation studies, but the choice of this research method was deliberate. For the purpose of the study, the most advanced neonatal simulator was used; in addition, applying the simulator enabled the randomized, cross-over study without any potential harm to the patient (16, 25). The second limitation was restricting the study group only to novice physicians; however, it is the medical personnel who have recently graduated from medical studies that should have up-to-date knowledge and skills in performing CPR in all age groups of patients.

## Conclusion

The median chest compression depth for nTTT and TTHT is significantly higher than that for TFT. The degree of full chest recoil was the highest for TFT, then for nTTT and TTHT. The effective compression efficiency with nTTT was the highest, followed by TTHT and TFT. Our novel newborn chest compression method applied in this manikin study provided adequate chest compression depth, full chest recoil, and acceptably effective compression efficiency. Further clinical studies are necessary to confirm these initial results.

## Author contributions

JS, LS, and KR: conceptualization; JS, LS, KR, and JL: data curation; LS and AR-N: formal analysis; JS, LS, and JL: investigation; JS, AR-N, KR: methodology; JS and LS: project administration; JS, LS, and KR: writing–original draft; JS, LS, JL, AR-N, and KR: writing–review and editing.

### Conflict of interest statement

The authors declare that the research was conducted in the absence of any commercial or financial relationships that could be construed as a potential conflict of interest.

## Supplementary material

Supplementary Video 1 Video of new chest compression technique.

Supplementary Table Comparison of chest compression outcome variables.

## References

[1] Jung WJ Hwang SO Kim HI Cha YS Kim OH Kim H. ‘Knocking-fingers' chest compression technique in infant cardiac arrest: single-rescuer manikin study. Eur J Emerg Med. (2018). 10.1097/MEJ.0000000000000539 [Epub ahead of print] 29384754

[2] Forrest A Butt WW Namachivayam SP. Outcomes of children admitted to intensive care after out-of-hospital cardiac arrest in Victoria, Australia. Crit Care Resusc. (2017) 19:150–8. 28651511

[3] Maconochie IK Bingham R Eich C López-Herce J Rodríguez-Núñez A Rajka T Paediatric life support section Collaborators. European Resuscitation Council Guidelines for Resuscitation 2015: section 6. paediatric life support. Resuscitation (2015) 95:223–48. 10.1016/j.resuscitation.2015.07.028 26477414

[4] de Caen AR Berg MD Chameides L Gooden CK Hickey RW Scott HF Part 12: pediatric advanced life support: 2015 American Heart Association Guidelines Update for cardiopulmonary resuscitation and emergency cardiovascular care. Circulation (2015) 132:526–42. 10.1161/CIR.0000000000000266 26473000 PMC6191296

[5] Houri PK Frank LR Menegazzi JJ Taylor R. A randomized, controlled trial of two-thumb vs two-finger chest compression in a swine infant model of cardiac arrest. Prehosp Emerg Care (1997) 1:65–7. 10.1080/10903129708958789 9709339

[6] Menegazzi JJ Auble TE Nicklas KA Hosack GM Rack L. Two-thumb versus two-finger chest compression during CRP in a swine infant model of cardiac arrest. Ann Emerg Med. (1993) 22:240–3. 10.1016/S0196-0644(05)80212-4 8427439

[7] Whitelaw CC Slywka B Goldsmith LJ. Comparison of a two-finger versus two-thumb method for chest compressions by healthcare providers in an infant mechanical model. Resuscitation (2000) 43:213–6. 10.1016/S0300-9572(99)00145-8 10711490

[8] Wyllie J Bruinenberg J Roehr CC Rüdiger M Trevisanuto D Urlesberger B. European resuscitation council guidelines for resuscitation 2015: section 7. Resuscitation and support of transition of babies at birth. Resuscitation (2015) 95:249–63. 10.1016/j.resuscitation.2015.07.029 26477415

[9] Wyllie J Perlman JM Kattwinkel J Wyckoff MH Aziz K Guinsburg R. Part 7: neonatal resuscitation: 2015 international consensus on cardiopulmonary resuscitation and emergency cardiovascular care science with treatment recommendations. Resuscitation (2015) 95:169–201. 10.1016/j.resuscitation.2015.07.045 26477424

[10] Jiang J Zou Y Shi W Zhu Y Tao R Jiang Y. Two-thumb-encircling hands technique is more advisable than 2-finger technique when lone rescuer performs cardiopulmonary resuscitation on infant manikin. Am J Emerg Med. (2015) 33:531–4. 10.1016/j.ajem.2015.01.025 25667159

[11] Smereka J Bielski K Ladny JR Ruetzler K Szarpak L. Evaluation of a newly developed infant chest compression technique: a randomized crossover manikin trial. Medicine (2017) 96:5915–9. 10.1097/MD.0000000000005915 28383397 PMC5411181

[12] Smereka J Szarpak L Rodríguez-Núñez A Ladny JR Leung S Ruetzler K. A randomized comparison of three chest compression techniques and associated hemodynamic effect during infant CPR: a randomized manikin study. Am J Emerg Med. (2017) 35:1420–5. 10.1016/j.ajem.2017.04.024 28433454

[13] Smereka J Kasinski M Smereka A Ładny JR Szarpak Ł. The quality of a newly developed infant chest compression method applied by paramedics: a randomised crossover manikin trial. Kardiol Pol. (2017) 75:589–95. 10.5603/KP.a2017.0015 28150278

[14] Smereka J Szarpak L Smereka A Leung S Ruetzler K. Evaluation of new two-thumb chest compression technique for infant CPR performed by novice physicians. A randomized, crossover, manikin trial. Am J Emerg Med. (2017) 35:604–9. 10.1016/j.ajem.2016.12.045 28040386

[15] Fakhraddin BZ Shimizu N Kurosawa S Sakai H Miyasaka K Mizutani S. New method of chest compression for infants in a single rescuer situation: thumb-index finger technique. J Med Dent Sci. (2011) 58:15–22. 23896782

[16] Ladny JR Smereka J Rodríguez-Núñez A Leung S Ruetzler K Szarpak L. Is there any alternative to standard chest compression techniques in infants? A randomized manikin trial of the new “2-thumb-fist” option. Medicine (2018) 97:e9386. 10.1097/MD.0000000000009386 29384839 PMC5805411

[17] Kurowski A Szarpak Ł Bogdanski Ł Zaśko P Czyzewski Ł. Comparison of the effectiveness of cardiopulmonary resuscitation with standard manual chest compressions and the use of TrueCPR and PocketCPR feedback devices. Kardiol Pol. (2015) 73:924–30. 10.5603/KP.a2015.0084 25985725

[18] Shultz JJ Mianulli MJ Gisch TM Coffeen PR Haidet GC Lurie KG. Comparison of exertion required to perform standard and active compression-decompression cardiopulmonary resuscitation. Resuscitation (1995) 29:23–31. 10.1016/0300-9572(94)00812-T 7784719

[19] Wyckoff MH Aziz K Escobedo MB Kapadia VS Kattwinkel J Perlman JM. Part 13: neonatal resuscitation: 2015 american heart association guidelines update for cardiopulmonary resuscitation and emergency cardiovascular care. Circulation (2015) 132:S543–60. 10.1161/CIR.0000000000000267 26473001

[20] Udassi JP Udassi S Theriaque DW Shuster JJ Zaritsky AL Haque IU. Effect of alternative chest compression techniques in infant and child on rescuer performance. Pediatr Crit Care Med. (2009) 10:328–33. 10.1097/PCC.0b013e31819886ab 19307812 PMC4088329

[21] Christman C Hemway RJ Wyckoff MH Perlman JM. The two-thumb is superior to the two-finger method for administering chest compressions in a manikin model of neonatal resuscitation. Arch Dis Child Fetal Neonatal Ed. (2011) 96:F99–101. 10.1136/adc.2009.180406 20847197

[22] Kaminska H Wieczorek W Matusik P Czyzewski L Ladny JR Smereka J. Factors influencing high quality chest compressions during cardiopulmonary resuscitation scenario according to 2015 American Heart Association Guidelines. Kardiol. Pol. (2018) 76:642–7. 10.5603/KP.a2018.0003 29313566

[23] Contri E Cornara S Somaschini A Dossena C Tonani M Epis F. Complete chest recoil during laypersons' CPR: Is it a matter of weight? Am J Emerg Med. (2017) 35:1266–8. 10.1016/j.ajem.2017.03.060 28377054

[24] Travers AH Perkins GD Berg RA Castren M Considine J Escalante R. Part 3: adult basic life support and automated external defibrillation: 2015 international consensus on cardiopulmonary resuscitation and emergency cardiovascular care science with treatment recommendations. Circulation (2015) 132:S51–83. 10.1161/CIR.0000000000000272 26472859

[25] Smereka J Szarpak L Smereka A Leung S Ruetzler K. New method of infant chest compression. Authors response. Am J Emerg Med. (2017) 35:795. 10.1016/j.ajem.2016.12.068 28062206

