# Supplementary material for: A Novel Method of Newborn Chest Compression: A Randomized Crossover Simulation Study
Source: Front Pediatr. 2018 May 29;6:159. doi: 10.3389/fped.2018.00159 (PMC5986950; doi:10.3389/fped.2018.00159)
Supplement: Supplementary Table — Comparison of chest compression outcome variables. [file Table_2.docx]

| Supplementary Table. Comparison of chest compression outcome variables | | | | |
| --- | --- | --- | --- | --- |
| **Variable** | **nTTT** | **TFT** | **TTHT** | **p-value** |
| Chest compression depth [mm] | 3.4  [3.4-3.6] | 2.5  [2.2-2.6] | 3.2  [3.2-3.5] | nTTT vs. TFT = **<0.001**  nTTT vs. TTHT = 0.101  TFT vs. TTHT = **<0.001** |
| Full release [%] | 93  [91-97] | 99  [96-100] | 90  [74-91] | nTTT vs. TFT = **<0.001**  nTTT vs. TTHT = **0.016**  TFT vs. TTHT = **<0.001** |
| Effective compression [%] | 96  [96-96] | 85  [82-88] | 86  [84-92] | nTTT vs. TFT = **<0.001**  nTTT vs. TTHT = **<0.001**  TFT vs. TTHT = 0.672 |
